# Emerging Neuroprotective Strategies: Unraveling the Potential of HDAC Inhibitors in Traumatic Brain Injury Management

**DOI:** 10.2174/1570159X22666240128002056

**Published:** 2024-01-29

**Authors:** Lisha Ye, Wenfeng Li, Xiaoyan Tang, Ting Xu, Guohua Wang

**Affiliations:** 1 Department of Neurophysiology and Neuropharmacology, Institute of Special Environmental Medicine and Co-Innovation Center of Neuroregeneration, Nantong University, 9 Seyuan Road, Chongchuan District, Nantong, Jiangsu 226019, China

**Keywords:** Traumatic brain injury, neuroprotective drugs, HDAC inhibitors, neuroinflammation, oxidative stress, neuronal apoptosis, axonal regeneration, glial activation

## Abstract

Traumatic brain injury (TBI) is a significant global health problem, leading to high rates of mortality and disability. It occurs when an external force damages the brain, causing immediate harm and triggering further pathological processes that exacerbate the condition. Despite its widespread impact, the underlying mechanisms of TBI remain poorly understood, and there are no specific pharmacological treatments available. This creates an urgent need for new, effective neuroprotective drugs and strategies tailored to the diverse needs of TBI patients. In the realm of gene expression regulation, chromatin acetylation plays a pivotal role. This process is controlled by two classes of enzymes: histone acetyltransferase (HAT) and histone deacetylase (HDAC). These enzymes modify lysine residues on histone proteins, thereby determining the acetylation status of chromatin. HDACs, in particular, are involved in the epigenetic regulation of gene expression in TBI. Recent research has highlighted the potential of HDAC inhibitors (HDACIs) as promising neuroprotective agents. These compounds have shown encouraging results in animal models of various neurodegenerative diseases. HDACIs offer multiple avenues for TBI management: they mitigate the neuroinflammatory response, alleviate oxidative stress, inhibit neuronal apoptosis, and promote neurogenesis and axonal regeneration. Additionally, they reduce glial activation, which is associated with TBI-induced neuroinflammation. This review aims to provide a comprehensive overview of the roles and mechanisms of HDACs in TBI and to evaluate the therapeutic potential of HDACIs. By summarizing current knowledge and emphasizing the neuroregenerative capabilities of HDACIs, this review seeks to advance TBI management and contribute to the development of targeted treatments.

## 1 INTRODUCTION

Traumatic brain injury (TBI) is a complex neurological condition resulting from external forces impacting the head, leading to brain dysfunction. It is typically estimated to affect nearly 69 million individuals annually [1]. Between 1990 and 2016, the global burden of TBI significantly increased, with the age-standardized incidence rate rising by 8.4%. TBI is increasingly considered a critical global health issue, as the overall incidence and prevalence of TBI are likely to increase in the future [2]. It is also considered a risk factor for late-onset of Alzheimer’s disease [3]. In the past twenty years, there’s been a significant advancement in our comprehension of the intricate pathophysiology of TBI [4]. However, despite myriad research efforts conducted on animal models of TBI in pursuit of therapeutic interventions, there is no effective drug for the treatment of brain-injured patients [5]. Thus, understanding and developing neuroprotective agents that inhibit posttraumatic cell death are importance [6]. Even though the current situation appears bleak, significant advancements in interconnected medical fields have been achieved in the past twenty years, fostering a more hopeful perspective towards developing new drugs for brain trauma [7]. The intricate regulation of gene expression is governed by a myriad of epigenetic mechanisms, among which histone modification stands as a pivotal player. Acetylation, orchestrated by the opposing actions of histone acetyltransferase (HAT) and histone deacetylase (HDAC), is just one of the many mechanisms that modulate histone modifications [8]. These enzymes compete to regulate the acetylation status of lysine residues in histones, thereby influencing gene expression in various biological processes [9]. Apart from acetylation, other significant epigenetic mechanisms include DNA methylation, non-coding RNA expression, and histone methylation, among others [10, 11]. Each of these mechanisms plays a unique role in fine-tuning gene expression, ensuring cellular function and response to environmental cues. In the realm of neurobiology, the balance of acetylation and deacetylation is especially crucial. Disruptions in this equilibrium, such as those caused by diminished HAT activity or augmented HDAC activity, have been implicated in various neuropathological conditions [12]. There's mounting evidence suggesting that neurodegeneration is often associated with a shift towards hyper-deacetylation stemming from these disruptions [13]. It is believed that HDAC inhibitors (HDACIs), as unique and generally effective neuroprotective agents, have the potential to radically improve the often bleak prognosis for brain trauma patients, thereby marking the first stride in tackling the silent epidemic of brain injury [14]. HDACIs have shown neuroprotective and neuroregenerative attributes in various brain disease animal models [15]. A significant amount of research has been directed toward studying HDACIs as innovative treatments in models of ischemic stroke [16], multiple sclerosis [17], Alzheimer’s disease [18], and Huntington’s disease [19]. Bearing this in mind, several studies scrutinizing the application of HDACIs for restoring histone acetylation and transcriptional activation in TBI models have been discussed [20-22]. The review is to present an overview of the mechanisms through which HDACIs regulate neuroprotection and to explore the implications of a new epigenetic approach to enhance neurological recovery post-TBI. We believe this knowledge will contribute to establishing a logical basis for the development of HDACIs as a treatment for TBI.

## 2 PATHOPHYSIOLOGY OF TBI

**Fig. (1):**
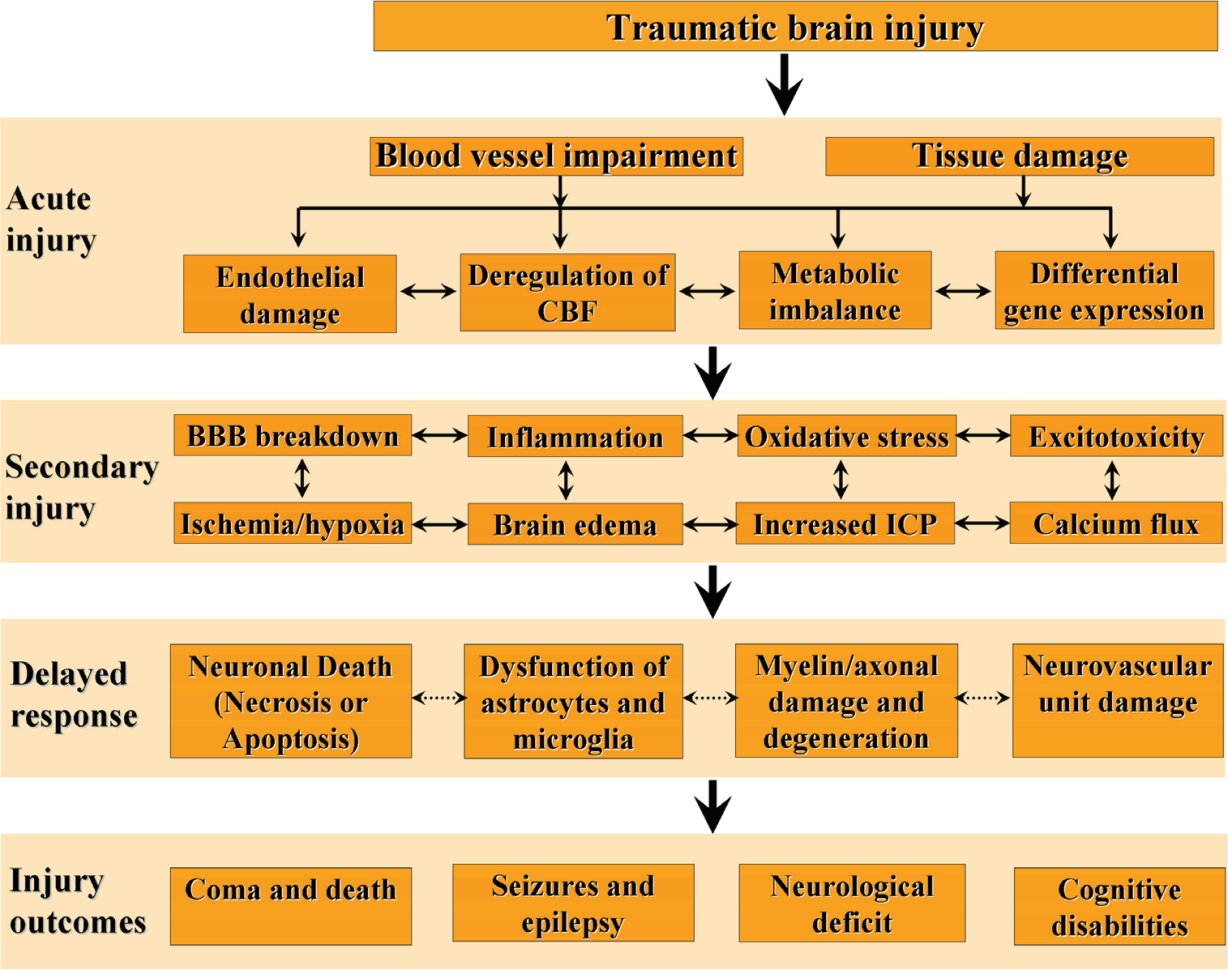
Pathophysiological cascades in traumatic brain injury. The injuries that occur as a result of TBI can be divided into two categories: primary and secondary injury. Primary brain injury happens instantaneously during the trauma, causing brain tissues and blood vessels to stretch, compress, and tear. This leads to endothelial damage, irregularities in blood flow, metabolic imbalances, and membrane perturbation. These processes may directly trigger signaling cascades and intricate interactions between pathological processes within the secondary injury phase. Secondary injury events encompass the breakdown of the blood-brain barrier, the release of inflammation-inducing factors, an overload of free radicals (oxidative stress), excessive release of the neurotransmitter glutamate (excitotoxicity), an influx of calcium, and the formation of both vasogenic and cytotoxic brain edema. Additional elements in secondary injury include ischemia (inadequate blood flow), cerebral hypoxia (insufficient oxygen in the brain), and elevated intracranial pressure (pressure within the skull). These events can evolve, interact, and trigger immediate complications and delayed pathophysiological mechanisms, leading to a wide range of consequences.

Based on both clinical and experimental evidence, it is clear that TBI does not only cause primary brain injury, which is more or less complete at the time of impact [23], but also produces a cascade of secondary brain injury induced by a complex interplay of cellular processes and biochemical cascades activated minutes to days after the initial trauma [24, 25]. Primary injury events following TBI, such as skull fractures, lacerations, hematomas, cerebral contusions, and diffuse axonal injury, are primarily responsible for irreversible brain damage [26, 27]. Secondary injuries, however, stem from various intra- and extracranial factors. Intracranial factors may include mass lesions, localized or widespread brain swelling, intracranial hypertension, seizures, vasospasm, or infection. Extracranial factors can comprise hypotension, hypoxia, hypercapnia or hypocapnia, hyperglycemia or hypoglycemia, anemia, fever, electrolyte imbalances, coagulopathy, and infection [28]. These secondary injuries resulting from TBI lead to alterations in cellular function and extend injury through mechanisms such as excitotoxicity, depolarization, disruption of calcium homeostasis, production of free radicals, disruption of the blood-brain barrier, edema formation, ischemic injury, intracranial hypertension [4, 29] (Fig. **1**). While prevention is the only measure for primary injury, the 'evolving' pathology of the delayed secondary damage phase offers a therapeutic window for intervention, and thus, the management of TBI primarily focuses on preventing and treating secondary brain injury [30, 31].

## 3 NEUROPROTECTIVE EFFECTS OF HDACIs ON TBI

The acetylation balance is greatly impaired during TBI conditions [32]. State-of-the art research activity has focused on HDACIs as novel therapeutics in models of TBI [33]. Additionally, HDACIs may also have multiple neuroprotective effects acting by molecular means [34, 35]. Herein, we discuss the use of reinstating the acetylation homeostasis to ameliorate TBI and summarize our current understanding of the overall functional value of such therapy on TBI. In addition, the neuroprotective effect of HDACIs on TBI is listed in Table **1**. The therapeutic potential of HDAC inhibitors in the context of TBI has garnered significant attention in recent years. These inhibitors have demonstrated robust neuroprotective effects, especially when administered shortly after the injury [36]. A pertinent question that arises is the duration of these benefits. Some studies suggest that HDAC inhibitors can provide long-lasting neuroprotective effects [37]. For instance, microglia-specific HDAC3 knockout not only reduced proinflammatory microglial responses but also elicited long-lasting improvement of white matter integrity and functional recovery after TBI [37]. However, the exact duration of these benefits and whether they are truly long-lasting remains a topic of ongoing research. Another critical aspect to consider is the administration regimen. While some studies indicate that a single administration can yield significant benefits, it remains unclear if multiple administrations are necessary to achieve sustained benefits over time [38]. While they have shown promise in alleviating TBI-induced secondary brain injury, including neurological deficits and cerebral edema [34], caution must be exercised due to potential adverse effects. Comprehensive understanding and further research into the optimal dosing regimen, duration of treatment, and potential side effects are crucial for the successful clinical translation of HDAC inhibitors in TBI treatment.

### 3.1 Histone

Histones are small basic proteins abundant in the amino acids lysine and arginine, forming the core of the nucleosome, which organizes and structures DNA into units [39]. Continuous units of this nucleosome result in chromatin, the packaging material for the entire human genome [40]. Histones are highly conserved and can be primarily classified into five major types: H1, H2A, H2B, H3, and H4 [41]. Of these, H2A, H2B, H3, and H4 participate in the formation of the chromatin’s essential sub-unit, the nucleosome. A single nucleosomal core particle consists of a DNA fragment (146 bp) encircling a histone octamer, which is composed of an H3-H4 tetramer and two H2A-H2B dimers [41]. Each nucleosome is separated from the next by an area known as linker DNA, which is stabilized by the H1 histone. H1 binds to each nucleosome and its neighboring linker DNA, aiding in the association of adjacent nucleosomes [42]. The four nucleosomal histones consist of two domains: the C-terminal domain located within the nucleosome core and the  N-terminal domain with lysine residues projecting out of the nucleosome. Among the four histone tails, H3 and H4 are subjected to several posttranslational modifications, including acetylation, phosphorylation, and methylation [43]. Therefore, histone proteins play a critical role not only in DNA packaging and chromosome stabilization but also in the essential regulation and expression of genes.

### 3.2 The Acetylation Homeostasis: Acetylation and Deacetylation of Histone Protein

Acetylation levels play an integral role in controlling transcriptional activity. When acetylation occurs, it contributes to the formation of an open chromatin structure, which subsequently allows the transcription machinery to gain access to promoters [44]. Conventionally, chromatin acetylation has been observed to be associated with active transcription, commonly referred to as euchromatin, whereas deacetylation is correlated with gene silencing [45].

The equilibrium of acetylation within chromatin is regulated by two opposing enzyme classes: histone acetyltransferases (HATs) and histone deacetylases (HDACs). These two enzyme classes are in constant competition to control the acetylation status of lysine residues within histones [46]. In a general sense, HATs operate by acetylating lysine groups in nuclear histones. This process neutralizes the histones' charges, leading to a more open and transcriptionally active chromatin structure [47]. Conversely, HDACs operate by selectively removing acetyl groups from the ε-amino groups of lysine situated near the amino terminals of core histone proteins, which leads to transcriptional repression [48].

HATs and HDACs function by adding or removing acetyl groups from lysine residues in histones and various transcription factors, which form an integral part of the transcription initiation complex. These two enzyme classes are typically found within sizable multiprotein complexes, which are situated within or proximate to euchromatin [49]. HATs work to modify core histone tails through acetylation, which leads to a more relaxed chromatin structure. This alteration, in conjunction with the acetylation of certain transcription factors, results in improved DNA accessibility, promoter binding, and gene expression [50]. Contrastingly, HDACs impede the transcription process by deacetylating these targets, leading to a more compact chromatin structure, referred to as heterochromatin [51]. In this manner, the HAT-HDAC system serves as a pivotal regulatory mechanism in controlling gene expression [49]. Augmentation of acetylation can be accomplished by inhibiting deacetylation, a process that can be performed with a variety of types of HDAC inhibitors [52].

### 3.3 HDAC and HDACIs

Research has identified 18 human genes thus far, all of which encode either established or potential HDACs [53]. The HDACs can be categorized into two distinct families and four sub-groups: the conventional family and the family of silent information regulator 2 (Sir2)-associated proteins, also known as sirtuins [54]. The conventional family consists of various HDACs, such as HDAC1, 2, 3, and 8, which belong to class I; HDAC4, 5, 6, 7, 9, and 10, which fall under class II; and HDAC11, which is classified under class IV [55]. A common trait across these enzymes is their sequence resemblance, and all necessitate Zn^2+^ to carry out their deacetylase activity. Contrastingly, the sirtuin family comprises seven members (SIRT1-7) falling under class III. These sirtuins do not share sequence similarity with members of the conventional family and require NAD^+^ to function as their cofactor [55].

Class I HDACs, which include HDAC1, 2, and 8, are primarily found within the nucleus [56]. HDAC3, however, is located both within the nucleus and the cytoplasm and is also known to be associated with the cellular membrane [57]. Unlike class I, which is strictly expressed within the nucleus, class II HDACs (HDAC4, 5, 6, 7, 9, and 10) are known to move between the nucleus and the cytoplasm [58]. Class III HDACs (SIRT1 - SIRT7), homologs of yeast Sir2, form a distinct structural group of enzymes that depend on NAD^+^ and are located both in the nucleus and cytoplasm [59]. HDAC11, belonging to class IV, exhibits traits characteristic of both class I and II HDACs [60].

Historically, HDACIs have been employed in psychiatry and neurology as mood stabilizers and anti-epileptics [61]. HDAC inhibition could potentially be protective by tipping the HAT-HDAC balance towards HAT activity, thus alleviating transcriptional repression [62]. Recently, efforts have been made to develop HDACIs for cancer therapy, with some FDA-approved for clinical treatment or under clinical trials [63]. The precise mechanisms through which these compounds may operate are not completely clear, but epigenetic pathways are suggested [64]. In newer developments, HDACIs have been identified as strong candidates for anti-inflammatory drugs, offering novel therapeutic approaches for conditions like rheumatoid arthritis or lupus erythematosus [65]. Furthermore, HDACIs are being investigated as neuroprotective agents for neurodegenerative diseases [66], stroke [67], and brain injury [68], exhibiting tremendous potential. HDACIs are recognized for their ability to target non-histone protein substrates that are integral in transcription, nuclear transport, cytoskeleton organization, and signal transduction. The acetylation status of these substrates can influence their stability and interactions with other proteins [69]. By modulating the acetylation degree of these molecules, HDACIs can increase or inhibit their activity, thereby potentially altering cellular behavior in beneficial ways.

### 3.4 The Underlying Mechanisms of HDACIs in the Treatment of TBI

Given a great deal of success, HDACIs have been used in experimental models of various neurodegenerative disorders. HDACIs, as novel and broadly effective neuroprotective agents, have also been targeted for the treatment of TBI [90]. Indeed, enhancement of histone acetylation by inhibition of HDAC is part of the mechanism underlying the beneficial effects of TBI [91]. Consistent with this, accumulating evidence has demonstrated that applying HDACIs following TBI may be an effective strategy to improve motor function, enhance neuronal plasticity and spatial memory, attenuate tissue damage, reduce the number of degenerating neurons, and lessen some of the pathologies associated with brain injury [18]. The neuroprotective attributes of HDACIs are associated with their capacity to reestablish appropriate acetylation levels, thereby amplifying the expression of genes that contribute to neuronal plasticity and survival [92]. Additionally, the inhibition of HDAC may stimulate the expression of anti-mitotic and anti-apoptotic genes, such as p21 and HSP-70, which foster cellular survival [93]. HDACIs also have the potential to influence other neural cell types within the central nervous system (CNS), including reactive astrocytes and microglia, thereby reducing inflammation and secondary damage during neuronal injury or disease [94]. Subsequently, this review is focused on summarizing the neuroprotective effect of HDACIs and the involved mechanisms on TBI.

#### 3.4.1 Reducing Neuroinflammatory Response

The growing interest in HDACIs as orally administered, safe, anti-inflammatory agents stems from their proven capability to mitigate disease severity in various animal models of inflammatory and autoimmune diseases [7, 17]. Evidence suggests that the pharmacological inhibition of HDAC can prevent the transcription of proinflammatory mediators [95], reduce cyclooxygenase 2 levels [96], and decrease the density of phagocytic microglia [97], all of which play a role in neuroinflammation [98]. Furthermore, neuroinflammatory responses, partly mediated by activated microglia - the resident immune cells of the CNS, are believed to significantly impact neuronal survival and brain function [99]. TBI also initiates a rapid and robust inflammatory response in the brain, partly characterized by the activation of microglia [100]. At low nanomolar concentrations, the pan-HDACI ITF2357 has shown potent anti-inflammatory effects both *in vitro* and *in vivo* [101]. In a head trauma model, it was demonstrated that the accumulation of lectin-positive microglia/macrophages within the ipsilateral hippocampus was reduced following post-injury administration of ITF2357 [82]. Similarly, HDACIs such as SAHA exhibit immunosuppressive and anti-inflammatory properties by decreasing cytokine production [102]. This, along with previous findings that DMA-PB (a novel HDACI) can mitigate microglial activation and inflammation following lateral fluid percussion injury in rats [89], suggest that pharmacological inhibition of HDAC could be a potential new therapeutic approach for inhibiting neuroinflammation associated with TBI. HDACIs have also been shown to significantly attenuate the inflammatory microglia response and potentially reduce neuronal degeneration in the hippocampus [103]. Additionally, HDAC inhibition can reduce inflammatory responses through cytokine synthesis regulation and by inducing apoptosis and/or reducing the proliferation of inflammatory cells [104].

Several mechanisms have been proposed for how HDACIs exert their anti-inflammatory effects. Firstly, they might inhibit the production of inflammatory cytokines and nitric oxide, both of which play crucial roles in the inflammatory process [105]. Secondly, HDACIs might also inhibit key transcription factors involved in inflammation, like NF-κB and STAT, thereby suppressing the expression of genes involved in the inflammatory response [106]. Finally, HDACIs may also modulate inflammation by inhibiting the proliferation or inducing differentiation of normal cells during inflammation [107]. The effective concentrations of HDACs for anti-inflammatory actions are in the low nanomolar range, which is substantially lower than the micromolar concentrations needed for their anticancer effects, where the mechanism is to increase apoptosis by upregulating the expression of proapoptotic genes [108]. This suggests that HDACIs could be employed at lower, safer doses in the context of TBI, potentially reducing the risk of side effects.

#### 3.4.2 Lessening Oxidative Stress

TBI is a complex pathological event, and its association with oxidative stress is well-documented. Following TBI, there is an immediate and pronounced increase in oxidative stress, which plays a pivotal role in driving neuronal dysfunction and subsequent cell death [109]. Given this relationship, therapeutic strategies that can counteract oxidative stress have the potential to offer significant benefits in the management of TBI, partly to our understanding of cellular defense mechanisms against oxidative stress followed by the vitagene network, a collection of genes that play a crucial part in the cellular response to stress, especially in the context of neurodegenerative disorders [110, 111]. Vitagenes, encompassing entities like heat shock proteins, thioredoxin, and sirtuins, are not merely bystanders in the stress response. Instead, they actively engage and respond to stressors, working to restore cellular balance and homeostasis. The importance of this dynamic response becomes evident when considering the consequences of unchecked oxidative stress. If not effectively managed, oxidative stress can precipitate a cascade of cellular events leading to damage, apoptosis, and, in the long run, degenerative diseases [112]. A particularly concerning aspect of this is the vulnerability of our cells' genetic material to oxidative damage, as highlighted by the tangible harm it can inflict on nuclear DNA [113]. Within this framework of oxidative stress and its implications, HDACIs present a promising avenue for intervention. Their capacity to shield neurons from oxidative stress-induced death underscores their potential therapeutic value, offering hope for more effective TBI treatments in the future [114].

HDACIs such as TSA, SAHA, and sodium butyrate have been shown to protect cultured primary cortical neurons from oxidative toxicity induced by glutathione depletion [114]. These inhibitors target multiple HDAC isoforms and have demonstrated promise in protecting neurons from oxidative stress-induced death. Interestingly, the inhibition of HDAC6, a specific isoform, has been linked to an increase in cellular antioxidant activity, suggesting a potential mechanism through which HDACIs may counteract oxidative insults.

HDACIs have also demonstrated neuroprotective effects against oxidative toxicity *in vivo* [114]. For example, sodium butyrate has been shown to ameliorate manic-like behavior and regulate antioxidant enzyme activity in rat models of mania, protecting the brain from oxidative damage [115]. Valproate, another HDACI, has been demonstrated to induce histone acetylation, enabling the transcription of enzymes that confer protection against oxidative stress [116-118]. In fact, valproate has been shown to ameliorate aluminum-induced oxidative stress and apoptosis in PC12 cells, a line of rat pheochromocytoma cells often used as a model for neuronal differentiation and neurodegeneration [119]. Valproate has also been found to enhance the expression of proteins related to endoplasmic reticulum stress, which can inhibit the accumulation of reactive oxygen species [120].

The cumulative impact of HDACIs on the brain's stress response may result from a complicated sequence of events related to both histone modification and the activation or inhibition of non-histone proteins dependent on hyperacetylation [121]. Evidence suggests that SAHA increases Hsp70 and Bcl-2 in the contralateral cortex of the ischemic brain, having pathophysiological implications [122]. Furthermore, the protection provided by HDACI in the oxidative stress model of death has been shown to partially require the transcription factor SP1 [123]. This is particularly noteworthy given the observation that the neuroprotection is blocked by Sp1 knockdown using Sp1-antisense oligodeoxynucleotides [124]. Indeed, the protective effects of HDACIs on oxidative stress-dependent neuronal death are underpinned by the activation of the Sp1 transcription factor dependent on hyperacetylation [125].

Research into Sp1's role in neuroprotection reveals that oxidative stress induces Sp1 acetylation, which bolsters DNA binding and the expression of Sp1-responsive reporter genes [125]. As a result, the neuroprotective effects provided by HDACIs, which amplify Sp1 acetylation, may be attributed to the activation of a collection of genes dependent on Sp1 that foster cellular survival [124, 125].

#### 3.4.3 Preventing Neuronal Apoptosis and Regulating Neurodegeneration

TBI triggers a series of pathological events, which include neuronal degeneration, tissue loss, compromised neuronal plasticity, and disruption of neurochemical regulation [126]. Neurodegeneration following TBI can affect several brain regions, including the cortex, hippocampus, and thalamus, and can occur in both the ipsilateral and contralateral hemispheres [127]. A primary aim of TBI treatment is to increase neuronal survival, as neurons are the most direct therapeutic target [6].

Emerging evidence suggests that transcriptional dysfunction, due to the misregulation of HAT and HDAC activity, may contribute to neurodegeneration during disease and injury [103]. Some studies have demonstrated an association between neurodegeneration and a global decrease in HAT activity, leading to relative hyper-deacetylation [101]. Thus, molecules that inhibit HDAC and thereby increase histone acetylation have significant potential as neuroprotective therapies. HDACIs, which are known to increase acetylation of histone-tails, have been demonstrated to aid learning and memory in not only wild-type mice but also in mouse models of neurodegeneration. This indicates the potentially significant role of HDACIs in mitigating cognitive deficits associated with neurodegenerative diseases [128].

Several HDAC inhibitors, including VPA, sodium butyrate, TSA, SAHA, and ITF2357, have been shown to decrease neuronal degeneration associated with TBI [73, 129]. These compounds have not only improved motor and cognitive performance but also reduced tissue damage. HDAC6 inhibition specifically has been reported to protect dopaminergic neurons from alpha-synuclein toxicity [130]. After a cortical impact injury in young rats, a decrease in acetylation of histone H3 is observed at 6 hours and 24 hours post-injury [131]. Additionally, the systemic administration of HDACIs in the acute phase has been linked with a potential reduction in hippocampal neurodegeneration following a lateral fluid percussion injury [132]. The observed decrease in neuronal degeneration, coupled with a significant restoration of histone acetylation levels post-treatment with HDACIs, suggests a possible link between these processes in the aftermath of TBI.

Axonal damage contributes to neuronal degeneration, with HDACs also implicated in this process [133]. SIRT1, a NAD-dependent histone deacetylase, has been shown to inhibit progressive neuronal damage after axonal transection in a specific mouse strain, demonstrating delayed Wallerian degeneration [134]. HDAC inhibition can potentially increase acetyl-tubulin and thereby enhance microtubule stability, delaying axonal degeneration [103]. Given the contribution of myelin loss to axonal damage and neurodegeneration in demyelinating disorders [135], HDAC inhibition presents a valuable therapeutic strategy for mitigating neurodegeneration following TBI.

Apoptosis, or programmed cell death, is another characteristic feature of neurodegeneration [136]. Furthermore, HDAC inhibitors have been demonstrated to exert a generalized neuroprotective action by inhibiting Bax-dependent neuronal apoptosis. This protective action is achieved through the selective repression of P53-target genes, thereby preventing the activation of Bax [137]. HDACIs have also been shown to prevent Bax-dependent caspase-3 cleavage, a mechanism that operates independently of P53 [138]. These findings suggest that HDACIs may shield neurons from various neurological insults by blocking both P53-dependent and P53-independent pathways. This gives a molecular insight into the neuroprotective action of HDAC inhibitors in neurons.

#### 3.4.4 Stimulating Neurogenesis and Promoting Axonal Regeneration

Neurogenesis refers to the complex process in which integrated neurons are formed from progenitor cells, a procedure involving cell proliferation, migration, and differentiation within the brain [139]. HDACs play a role in this process by facilitating chromatin compaction, which leads to gene transcription silencing and the regulation of neurogenesis [140]. In contrast, HDACIs function as vital regulators of neuronal differentiation at the transcriptional level [141]. Experiments involving the application of HDACIs to hippocampal neuronal progenitor cells have indicated that these can induce neural morphology and the expression of neuronal markers [142]. Scriptaid, an HDACI, has been shown to increase neuronal survival and the quantity and length of neuronal processes in the CA3 area of the hippocampus and the pericontusional cortex following TBI [70]. Moreover, treatment with Scriptaid appears to prevent traumatic brain injury (TBI)-induced reduction of phospho-AKT (p-AKT) and phosphorylated phosphatase and tensin homolog deleted on chromosome 10 (p-PTEN) in both cortical and CA3 hippocampal neurons [70]. Valproate, another HDACI, has shown promise in enhancing axonal regeneration and neuronal survival against various insults [143]. It seems to promote ERK pathway-dependent neuronal growth and attenuate neuronal apoptosis in rats with subarachnoid hemorrhage [144]. This suggests the potential utility for valproate in neurotrophic treatments for brain trauma, ischemia, and neurodegenerative diseases [144]. The influence of HDACIs extends to the transcriptional regulation of neuronal differentiation. For example, valproate-induced hippocampal neurogenesis has been associated with H4 acetylation and the Ngn1 promoter in hippocampal extracts [145]. The discovered link between HDACIs and the Ngn1, Math1, and p15 promoters further emphasizes the critical role of HDAC inhibition as a central regulator of neuronal differentiation at the transcriptional level [145]. In addition to these findings, VPA has been found to potentially enhance sciatic nerve regeneration by increasing the total number of regenerated myelinated nerve fibers in adult rats. This implies that VPA might be employed to promote or improve axonal regeneration in both the central and peripheral nervous systems in humans [146].

Neurite outgrowth is directed by a combination of extrinsic and intrinsic factors, which involve transcriptional regulation [147]. The lack of specific gene transcription appears to contribute to the failure of axon re-growth following injury in the CNS [148].The acetylation of histones and transcription factors, a process that enhances promoter accessibility and stimulates transcription, depends on the balance between the activities of HDACs and HATs. HDACs have been demonstrated to possess neuroprotective and neuroregenerative properties [149]. For instance, chronic administration of sodium butyrate was observed to increase overall histone acetylation and trigger neuroregeneration in CK-p25 mice [150]. Gaub and colleagues provided compelling evidence supporting the beneficial role of HDAC inhibition, as well as CBP/p300 and P/CAF acetylation-dependent transcriptional pathways, in neuronal outgrowth [151]. This suggests that enhancing neuronal acetylation and facilitating transcription through HDAC inhibition, as well as specific histone acetylating enzymes, may promote neuronal outgrowth under both physiological conditions and inhibitory substrates [151].

Collectively, these findings highlight the importance of transcriptional regulation in neuronal outgrowth and suggest potential strategies for promoting axonal regeneration after injury. By selectively targeting HDACs with pharmacological inhibitors, both neurogenesis and axonal regeneration can be promoted. These findings suggest that specific HDACs may be critical mediators of these neuroprotective and neuroregenerative effects.

#### 3.4.5 Reducing Glial Accumulation and Activation

HDACIs are capable of acting on a variety of cell types beyond neurons that are implicated in the pathology of TBI [22]. Glial cells, including astrocytes and microglia, undergo significant changes in response to neurotrauma, becoming “reactive” and contributing to both short and long-term pathological processes [152]. In the short term, reactive astrocytes can amplify pro-inflammatory responses, increase the permeability of the blood-brain barrier (BBB), and exacerbate cerebral edema, thus worsening neurological outcomes [153]. Over the long term, reactive astrocytes contribute to the formation of glial scars, a process known as astrogliosis, which can impair axonal regeneration and synaptic plasticity, thereby hindering recovery following TBI [154]. Various molecules involved in the inflammatory response and astrogliosis, such as GFAP, COX-2, iNOS, NO, IL-6, and TNF-α, can potentially be modulated by HDAC inhibition to attenuate these processes [155, 156].

Research suggests that the HDACI VPA can correct aberrant gene transcription and decrease reactive astrogliosis in a Yorkshire swine model of TBI [73]. Similarly, Scriptaid, another HDACI, has been shown to reduce glial accumulation and activation when administered 12 h post-TBI, and also decrease neuronal degeneration at the injury site [70, 71]. ITF2357, another HDACI, has been reported to decrease not only microglial accumulation following injury, but also the number and activation level of astrocytes. This effect appears to be due, at least in part, to ITF2357 promoting apoptosis of microglial cells, thus enhancing their clearance from the injury site and reducing gliosis [82]. Given that ITF2357 promotes the clearance of activated microglia/macrophages from the trauma penumbra through apoptosis, such a mechanism would also lead to reduced gliosis. The study by Prozorovski and colleagues unveiled that activating the HDAC Sirt1 could inhibit the proliferation of mouse neural progenitor cells and guide their differentiation towards the astroglial lineage, reducing the neuronal lineage. Interestingly, the application of reducing conditions resulted in a contrasting effect [157]. Subsequently, it was found that the knockdown of Sirt1 by shRNA or HDAC inhibitors prevented oxidation-mediated suppression of neurogenesis and reactive astrogliosis [157].

The prospect of inducing an apoptotic effect on glial cells might provide a therapeutic avenue for managing conditions like glioblastomas [158]. It could also help address astrogliosis following TBI, leading to the resolution of dysfunction in astrocytes and microglia [159]. Highly effective HDAC inhibitors are viewed as potential enhancements to current chemotherapy protocols. They work through epigenetic pathways to instigate apoptosis and enhance the shrinkage and subsequent regression of tumors [160]. HDACIs have been observed to interfere with the cell cycle at the G2 phase, compelling cells to enter the M phase prematurely. They also directly interfere with the mitotic spindle checkpoint [161]. Notably, HDAC inhibitors appear to be more successful at inducing cell cycle arrest and apoptosis in cancerous or reactive cells than in non-transformed cells. However, the underlying mechanisms responsible for this phenomenon are not yet entirely comprehended [162]. Indeed, it has been found that HDACIs promote neuronal survival and prevent neuronal apoptosis in excitotoxic and hypoxic conditions [163]. Thus, the varied effects of HDACIs on cell survival and death are challenging to explain and may be due to differences in cell types and cellular injuries or to the diverse structures of HDACIs. The contrasting effects of HDACIs across various paradigms can be explained by the tissue and stage-specific expression of different classes of HDACs [164]. On the other hand, it is generally believed that HDACIs differentially alter the balance of expression between pro- and anti-apoptotic genes in response to various biological demands [138]. An alternate strategy could be the use of broad-spectrum pharmacological inhibition that might target different HDAC isoforms found in the nucleus or cytoplasm. These isoforms have distinct non-histone substrates like transcription factors or cytoskeletal proteins [103]. In conclusion, the detailed mechanism of how HDACIs influence apoptosis still needs further elucidation.

## CONCLUSION

**Fig. (2):**
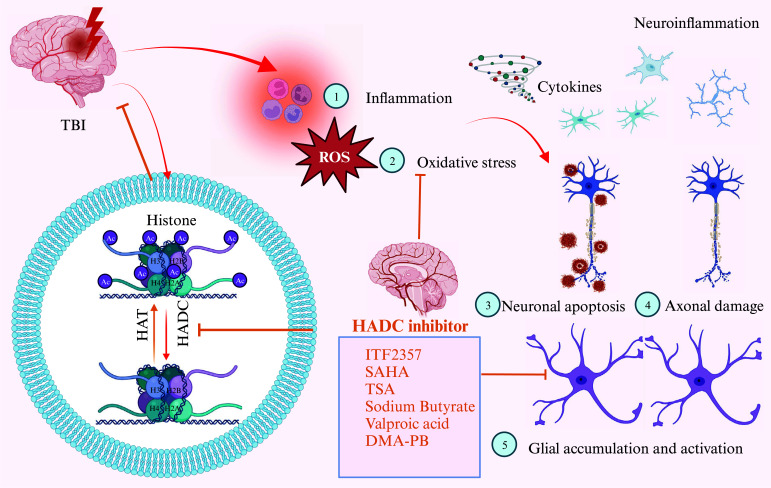
The potential neuroprotective effects of HDACIs on TBI. The lysine-rich tails of histone proteins undergo acetylation and deacetylation, processes facilitated by histone acetylase (HAT) and histone deacetylase (HDAC), respectively. A decrease in HAT function or an increase in HDAC activity can lead to reduced acetylation of histones and transcription factors, resulting in transcriptional repression. This imbalance may be a fundamental factor in the neurological damage associated with traumatic brain injury (TBI). By inhibiting HDAC, the balance between HAT and HDAC may be tipped in favour of HAT activity, thereby alleviating transcriptional repression. Furthermore, HDAC inhibition could trigger a range of neuroprotective effects, thereby mitigating secondary damage following neurotrauma. Image created with BioRender.com with permission.

The urgent need to develop new therapeutics for TBI treatment, coupled with the current absence of specific therapies for this condition, underscores the critical importance of identifying and characterizing innovative therapeutic candidates. The findings presented here provide a crucial link in evaluating HDACIs as potential novel agents for preventing and mitigating acute CNS injury in animal models, thereby warranting further exploration for TBI treatment. Beyond compensating for the loss of HAT activity and restoring appropriate acetylation levels, several observations from the above studies suggest that HDACIs also trigger multiple mechanisms that significantly contribute to neuroprotection (Fig. **2**). However, additional research is necessary to ascertain the full extent of the benefits provided by HDACIs and to clarify the genes and molecular targets of their neuroprotective actions, areas where experimental evidence remains insufficient.

## Figures and Tables

**Table 1:** Neuroprotective effect of HDACIs on TBI.

**HDAC ** **Inhibitor**	**Targets**	**Species**	**Experimental Model**	**Effects and Mechanisms**	**Drug Dose**	**Administration ** **Strategy**	**References**
Scriptaid	HDAC	Mice	CCI model	Scriptaid protects against TBI *via* modulation of PTEN and AKT pathway	1.5-5.5 mg/kg	i.p. at 12 hours after injury for 2 days	[70]
	HDAC	Mice	CCI model	Scriptaid prevents white matter injury by modulating microglia/macrophage polarization through the GSK3β/PTEN/Akt	3.5 mg/kg	i.p. at 2 hours after TBI for 2 days	[71]
	HDAC1, 2, 3	Rats	Neonatal rat HBID model	Scriptaid inhibits inflammatory responses and provides protection to the brain due to the promotion of microglia polarization in brain tissue toward the M2 phenotype	3.5 mg/kg	i.p. after HBID for 7 days	[72]
LB-205	HDAC	Rats	TBI model	LB-205 increases nestin in the damaged repair area, maintains the full expression of nerve growth factor (NGF) and the activation of neurotrophic tyrosine kinase receptor type 1 (TrkA) pathway, and reduces the expression of GFAP	10 mg/kg	i.p. at 4 hours, 24 hours, and 48 hours after TBI	[22]
CI-994	HDAC2	Mice	CCI model	CL-994 enhances BDNF and promotes neuronal rewiring and functional recovery following TBI	10 mg/kg	i.p. at day 28 post-injury for 14 days	[34]
Valproic acid (VPA)	HDAC	Swine	TBI model	VPA decreases neural apoptosis, inflammation, and degenerative changes and promotes neural plasticity in a long-term model of TBI *via* the regulation of NF-κB and IκB-α pathways	150 mg/kg	i.v. at 1 hour after injury for 3 hours	[73]
		Swine	TBI+HS model	VPA down-expresses nuclear factor-κB (NF-κB)-mediated cytokines, TYROBP, TREM2, CCR1, and IL-1β, and regulates the inflammatory expression in the model of TBI+HS	300 mg/kg	i.v. at 2 hours after injury for 6 hours	[74]
		Swine	TBI model	VPA demonstrates remarkable efficacy in enhancing neurologic recovery and reducing brain lesion size in swine subjected to hemorrhagic shock and TBI	50,150 mg/kg	i.v. at 2 hours after injury	[75]
		Rats	TBI model	VPA significantly reduces hippocampal dendritic damage associated with TBI and minimizes cortical contusion volume, leading to improvements in motor function and spatial memory	400 mg/kg	i.p. at 30 minutes or 3 hours after injury for 5 days every 24 hours	[76]
		Swine	TBI+HS model	VPA triggers metabolic alterations in the brain within the initial hours following a traumatic brain injury, thereby fostering a neuroprotective environment	150 mg/kg	i.v. after injury for 6 hours	[77]
		Swine	TBI model	VPA reduces serum glial fibrillary protein and affects the calcium signaling pathway after TBI, including mitochondrial metabolism and biosynthesis mechanisms, and plays neuroprotective and pro-survival roles.	150 mg/kg	i.v. at 1 hour after injury over 1 hour	[33]
		Rats	TBI model	VPA enhances glycocalyx shedding and decreases the volume of lesion size in injured animals	300 mg/kg	i.v. at 30 minutes after TBI for 5 minutes	[78]
		Swine	TBI+HS model	VPA modifies the early transcription of pathways associated with cell survival following TBI	100 mg/kg	i.v. at 2 hours after injury for 6 hours	[79]
		Swine	TBI+HS model	VPA fosters a conducive environment for the generation of new neurons, the elimination of damaged cells, and the reduction of inflammation	100 mg/kg	i.v. at 2 hours after injury for 6 hours	[80]
		Swine	TBI+HS model	VPA enhances the Rho GTPase signaling pathway and subsequently strengthens pro-cytoskeletal stability networks	150 mg/kg	i.v. at 1 hour after injury over 90 minutes	[81]
		Swine	TBI+HS model	VPA enhances platelet activation in both serum and brain tissue, which could potentially reduce lesion size and provide protection following TBI and hemorrhagic shock	300 mg/kg	i.v. at 2 hours after injury for 6 hours	[79]
ITF2357	HDAC	Mice	CHI model	ITF2357 enhances functional recovery and triggers glial apoptosis following experimental TBI	10 mg/kg	i.p. at 30 minutes prior to the injury, 1 or 24 hours after trauma	[82]
Trichostatin A (TSA)	HDAC	Rats	rMTBI	TSA rectifies the deficits in recognition memory and HDAC activities induced by rMTBI, accompanied by corresponding changes in the levels of H3-K9ac and CART mRNA.	10 mg/kg	i.p. daily for three days (each injection 24 hours apart on the 28^th^, 29^th^ and 30^th^ day)	[83]
Vorinostat (SAHA)	HDAC	Rats	TBI model	SAHA blocks the IL-23/IL-17 axis, thereby reducing neuronal apoptosis and improving the neural function recovery	12.5 mg/kg	i.v. Daily at 6 hours, 1, 3, and 7 days after TBI	[84]
		Rats	LFP rat model	Administering SAHA immediately after TBI results in an extended duration of hind paw sensitization compared to TBI without any treatment	50 mg/kg	i.p. after injury for 10 days	[21]
		Mice	Weight-drop model	SAHA mitigates TBI by activating the iNOS/ Nrf2/ARE pathway. It also protects against neuronal injury in TBI mice by reducing the altered levels of oxidative stress and inflammatory response	100 mg/kg	i.p. at 30 minutes before TBI	[85]
Sodium butyrate (SB)	HDAC	Rats	rMTBI model	SB rectifies the deficits induced by rMTBI in the acetylation levels of H3-K9 and CBP occupancy at the NPY promoter, thereby restoring both NPY expression and food intake	500 mg/kg	i.p. daily for 6 consecutive days from the 25^th^ to the 30^th^ day after the last trauma	[86]
		Mice	TBI model	sodium butyrate improves learning and memory in brain-injured mice	1.2 g/kg	i.p. at 7 days after injury for 4 weeks	[87]
ACY-1083	HDAC6	Swine	TBI+HS model	ACY-1083 dose-dependently suppresses the expression of hypoxia-inducible factor-1α while upregulating the phosphorylated mammalian target of rapamycin and heat shock protein 70. This leads to a reduction in lesion size and brain swelling following traumatic brain injury and hemorrhagic shock	30 mg/kg	i.v. after injury for 6 hours	[88]
DMA-PB	HDAC6	Rats	TBI model	DMA-PB enhances histone H3 acetylation and diminishes the inflammatory response of microglia following traumatic brain injury, leading to a potential trend toward decreased neuronal degeneration in the hippocampus	0, 0.25, 2.5, 25 mg/kg	i.p. after injury	[89]

**Abbreviations:** CCI: controlled cortical impact; HBID: hypoxia-ischemia brain damage; TBI: traumatic brain injury; TBI+HS: traumatic brain injury and hemorrhagic shock; CHI: closed head injury; rMTBI: repeated mild traumatic brain injury; LEP: lateral fluid percussion; i.v.: Intravenous injection; i.p.: Intraperitoneal injection.

## LIST OF ABBREVIATIONS

BBB: Blood-brain Barrier
CNS: Central Nervous System
HAT: Histone Acetyltransferase
HDAC: Histone Deacetylase
HDACIs: HDAC Inhibitors
TBI: Traumatic Brain Injury

## References

[1] Dewan M.C., Rattani A., Gupta S., Baticulon R.E., Hung Y.C., Punchak M., Agrawal A., Adeleye A.O., Shrime M.G., Rubiano A.M., Rosenfeld J.V., Park K.B. (2019). Estimating the global incidence of traumatic brain injury.. J. Neurosurg..

[2] Badhiwala J.H., Wilson J.R., Fehlings M.G. (2019). Global burden of traumatic brain and spinal cord injury.. Lancet Neurol..

[3] Schneider A.L.C., Selvin E., Latour L., Turtzo L.C., Coresh J., Mosley T., Ling G., Gottesman R.F. (2021). Head injury and 25‐year risk of dementia.. Alzheimers Dement..

[4] Kaur P., Sharma S. (2018). Recent advances in pathophysiology of traumatic brain injury.. Curr. Neuropharmacol..

[5] McGuire J.L., Ngwenya L.B., McCullumsmith R.E. (2019). Neurotransmitter changes after traumatic brain injury: An update for new treatment strategies.. Mol. Psychiatry.

[6] Akamatsu Y., Hanafy K.A. (2020). Cell death and recovery in traumatic brain injury.. Neurotherapeutics.

[7] Kalra S., Malik R., Singh G., Bhatia S., Al-Harrasi A., Mohan S., Albratty M., Albarrati A., Tambuwala M.M. (2022). Pathogenesis and management of traumatic brain injury (TBI): Role of neuroinflammation and anti-inflammatory drugs.. Inflammopharmacology.

[8] Park S.Y., Kim J.S. (2020). A short guide to histone deacetylases including recent progress on class II enzymes.. Exp. Mol. Med..

[9] Demyanenko S., Sharifulina S. (2021). The role of post-translational acetylation and deacetylation of signaling proteins and transcription factors after cerebral ischemia: facts and hypotheses.. Int. J. Mol. Sci..

[10] Irfan J., Febrianto M.R., Sharma A., Rose T., Mahmudzade Y., Di Giovanni S., Nagy I., Torres-Perez J.V. (2022). DNA Methylation and Non-Coding RNAs during tissue-injury associated pain.. Int. J. Mol. Sci..

[11] Dolinar A., Ravnik-Glavač M., Glavač D. (2018). Epigenetic mechanisms in amyotrophic lateral sclerosis: A short review.. Mech. Ageing Dev..

[12] Kabir F., Atkinson R., Cook A.L., Phipps A.J., King A.E. (2023). The role of altered protein acetylation in neurodegenerative disease.. Front. Aging Neurosci..

[13] Chatterjee S., Cassel R., Schneider-Anthony A., Merienne K., Cosquer B., Tzeplaeff L., Halder Sinha S., Kumar M., Chaturbedy P., Eswaramoorthy M., Le Gras S., Keime C., Bousiges O., Dutar P., Petsophonsakul P., Rampon C., Cassel J.C., Buée L., Blum D., Kundu T.K., Boutillier A.L. (2018). Reinstating plasticity and memory in a tauopathy mouse model with an acetyltransferase activator.. EMBO Mol. Med..

[14] Rodrigues D.A., Pinheiro P.S.M., Sagrillo F.S., Bolognesi M.L., Fraga C.A.M. (2020). Histone deacetylases as targets for the treatment of neurodegenerative disorders: Challenges and future opportunities.. Med. Res. Rev..

[15] Ziemka-Nalecz M., Jaworska J., Sypecka J., Zalewska T. (2018). Histone deacetylase inhibitors: A therapeutic key in neurological disorders?. J. Neuropathol. Exp. Neurol..

[16] Matheson R., Chida K., Lu H., Clendaniel V., Fisher M., Thomas A., Lo E.H., Selim M., Shehadah A. (2020). Neuroprotective effects of selective inhibition of histone deacetylase 3 in experimental stroke.. Transl. Stroke Res..

[17] Sun L., Telles E., Karl M., Cheng F., Luetteke N., Sotomayor E.M., Miller R.H., Seto E. (2018). Loss of HDAC11 ameliorates clinical symptoms in a multiple sclerosis mouse model.. Life Sci. Alliance.

[18] Nakatsuka D., Izumi T., Tsukamoto T., Oyama M., Nishitomi K., Deguchi Y., Niidome K., Yamakawa H., Ito H., Ogawa K. (2021). Histone Deacetylase 2 knockdown ameliorates morphological abnormalities of dendritic branches and spines to improve synaptic plasticity in an APP/PS1 Transgenic Mouse Model.. Front. Mol. Neurosci..

[19] Macabuag N., Esmieu W., Breccia P., Jarvis R., Blackaby W., Lazari O., Urbonas L., Eznarriaga M., Williams R., Strijbosch A., Van de Bospoort R., Matthews K., Clissold C., Ladduwahetty T., Vater H., Heaphy P., Stafford D.G., Wang H.J., Mangette J.E., McAllister G., Beaumont V., Vogt T.F., Wilkinson H.A., Doherty E.M., Dominguez C. (2022). Developing HDAC4-Selective protein degraders to investigate the role of hdac4 in huntington’s disease pathology.. J. Med. Chem..

[20] Lu J., Frerich J.M., Turtzo L.C., Li S., Chiang J., Yang C., Wang X., Zhang C., Wu C., Sun Z., Niu G., Zhuang Z., Brady R.O., Chen X. (2013). Histone deacetylase inhibitors are neuroprotective and preserve NGF-mediated cell survival following traumatic brain injury.. Proc. Natl. Acad. Sci. USA.

[21] Liang D.Y., Sahbaie P., Sun Y., Irvine K.A., Shi X., Meidahl A., Liu P., Guo T.Z., Yeomans D.C., Clark J.D. (2017). TBI-induced nociceptive sensitization is regulated by histone acetylation.. IBRO Rep..

[22] Lu J., Frerich J.M., Turtzo L.C., Li S., Chiang J., Yang C., Wang X., Zhang C., Wu C., Sun Z., Niu G., Zhuang Z., Brady R.O., Chen X. (2013). Histone deacetylase inhibitors are neuroprotective and preserve NGF-mediated cell survival following traumatic brain injury.. Proc. Natl. Acad. Sci..

[23] Sorby-Adams A., Marcoionni A., Dempsey E., Woenig J., Turner R. (2017). The role of neurogenic inflammation in blood-brain barrier disruption and development of cerebral oedema following acute central nervous system (CNS) injury.. Int. J. Mol. Sci..

[24] Hanscom M., Loane D.J., Shea-Donohue T. (2021). Brain-gut axis dysfunction in the pathogenesis of traumatic brain injury.. J. Clin. Invest..

[25] Salehi A., Zhang J.H., Obenaus A. (2017). Response of the cerebral vasculature following traumatic brain injury.. J. Cereb. Blood Flow Metab..

[26] Nikolian V.C., Dekker S.E., Bambakidis T., Higgins G.A., Dennahy I.S., Georgoff P.E., Williams A.M., Andjelkovic A.V., Alam H.B. (2018). Improvement of blood-brain barrier integrity in traumatic brain injury and hemorrhagic shock following treatment with valproic acid and fresh frozen plasma.. Crit. Care Med..

[27] Winkler E.A., Minter D., Yue J.K., Manley G.T. (2016). Cerebral edema in traumatic brain injury.. Neurosurg. Clin. N. Am..

[28] Vella M.A., Crandall M.L., Patel M.B. (2017). Acute management of traumatic brain injury.. Surg. Clin. North Am..

[29] Shi M., Chen F., Chen Z., Yang W., Yue S., Zhang J., Chen X. (2021). Sigma-1 Receptor: A potential therapeutic target for traumatic brain injury.. Front. Cell. Neurosci..

[30] Sande A., West C. (2010). Traumatic brain injury: A review of pathophysiology and management.. J. Vet. Emerg. Crit. Care (San Antonio).

[31] Desai M., Jain A. (2018). Neuroprotection in traumatic brain injury.. J. Neurosurg. Sci..

[32] Saha P., Gupta R., Sen T., Sen N. (2019). Histone deacetylase 4 downregulation elicits post-traumatic psychiatric disorders through impairment of neurogenesis.. J. Neurotrauma.

[33] Biesterveld B.E., Pumiglia L., Iancu A., Shamshad A.A., Remmer H.A., Siddiqui A.Z., O’Connell R.L., Wakam G.K., Kemp M.T., Williams A.M., Pai M.P., Alam H.B. (2020). Valproic acid treatment rescues injured tissues after traumatic brain injury.. J. Trauma Acute Care Surg..

[34] Sada N., Fujita Y., Mizuta N., Ueno M., Furukawa T., Yamashita T. (2020). Inhibition of HDAC increases BDNF expression and promotes neuronal rewiring and functional recovery after brain injury.. Cell Death Dis..

[35] Pumiglia L., Williams A.M., Kemp M.T., Wakam G.K., Alam H.B., Biesterveld B.E. (2021). Brain proteomic changes by histone deacetylase inhibition after traumatic brain injury.. Trauma Surg. Acute Care Open.

[36] Kim H.J., Rowe M., Ren M., Hong J.S., Chen P.S., Chuang D.M. (2007). Histone deacetylase inhibitors exhibit anti-inflammatory and neuroprotective effects in a rat permanent ischemic model of stroke: multiple mechanisms of action.. J. Pharmacol. Exp. Ther..

[37] Zhao Y., Mu H., Huang Y., Li S., Wang Y., Stetler R.A., Bennett M.V.L., Dixon C.E., Chen J., Shi Y. (2022). Microglia-specific deletion of histone deacetylase 3 promotes inflammation resolution, white matter integrity, and functional recovery in a mouse model of traumatic brain injury.. J. Neuroinflammation.

[38] Chen X., Wang H., Zhou M., Li X., Fang Z., Gao H., Li Y., Hu W. (2018). Valproic acid attenuates traumatic brain injury-induced inflammation in vivo: Involvement of autophagy and the Nrf2/ARE Signaling Pathway.. Front. Mol. Neurosci..

[39] Bowman G.D., Poirier M.G. (2015). Post-translational modifications of histones that influence nucleosome dynamics.. Chem. Rev..

[40] Bannister A.J., Kouzarides T. (2011). Regulation of chromatin by histone modifications.. Cell Res..

[41] Luger K., Dechassa M.L., Tremethick D.J. (2012). New insights into nucleosome and chromatin structure: an ordered state or a disordered affair?. Nat. Rev. Mol. Cell Biol..

[42] Fyodorov D.V., Zhou B.R., Skoultchi A.I., Bai Y. (2018). Emerging roles of linker histones in regulating chromatin structure and function.. Nat. Rev. Mol. Cell Biol..

[43] Nunez-Vazquez R., Desvoyes B., Gutierrez C. (2022). Histone variants and modifications during abiotic stress response.. Front. Plant Sci..

[44] Zovkic I.B., Paulukaitis B.S., Day J.J., Etikala D.M., Sweatt J.D. (2014). Histone H2A.Z subunit exchange controls consolidation of recent and remote memory.. Nature.

[45] Allis C.D., Jenuwein T. (2016). The molecular hallmarks of epigenetic control.. Nat. Rev. Genet..

[46] Shen Y., Wei W., Zhou D.X. (2015). Histone acetylation enzymes coordinate metabolism and gene expression.. Trends Plant Sci..

[47] Dang F., Wei W. (2022). Targeting the acetylation signaling pathway in cancer therapy.. Semin. Cancer Biol..

[48] Ramaiah M.J., Tangutur A.D., Manyam R.R. (2021). Epigenetic modulation and understanding of HDAC inhibitors in cancer therapy.. Life Sci..

[49] Chen R., Zhang M., Zhou Y., Guo W., Yi M., Zhang Z., Ding Y., Wang Y. (2020). The application of histone deacetylases inhibitors in glioblastoma.. J. Exp. Clin. Cancer Res..

[50] Ding P., Ma Z., Liu D., Pan M., Li H., Feng Y., Zhang Y., Shao C., Jiang M., Lu D., Han J., Wang J., Yan X. (2022). Lysine Acetylation/Deacetylation modification of immune-related molecules in cancer immunotherapy.. Front. Immunol..

[51] Filippakopoulos P., Knapp S. (2014). Targeting bromodomains: Epigenetic readers of lysine acetylation.. Nat. Rev. Drug Discov..

[52] Xue J., Wu G., Ejaz U., Akhtar F., Wan X., Zhu Y., Geng A., Chen Y., He S. (2021). A novel histone deacetylase inhibitor LT-548-133-1 induces apoptosis by inhibiting HDAC and interfering with microtubule assembly in MCF-7 cells.. Invest. New Drugs.

[53] Wang P., Wang Z., Liu J. (2020). Correction to: Role of HDACs in normal and malignant hematopoiesis.. Mol. Cancer.

[54] Bahl S., Seto E. (2021). Regulation of histone deacetylase activities and functions by phosphorylation and its physiological relevance.. Cell. Mol. Life Sci..

[55] Dewanjee S., Vallamkondu J., Kalra R.S., Chakraborty P., Gangopadhyay M., Sahu R., Medala V., John A., Reddy P.H., De Feo V., Kandimalla R. (2021). The Emerging Role of HDACs: Pathology and therapeutic targets in diabetes mellitus.. Cells.

[56] Kelly R.D.W., Cowley S.M. (2013). The physiological roles of histone deacetylase (HDAC) 1 and 2: Complex co-stars with multiple leading parts.. Biochem. Soc. Trans..

[57] Ferguson B.S., McKinsey T.A. (2015). Non-sirtuin histone deacetylases in the control of cardiac aging.. J. Mol. Cell. Cardiol..

[58] Wang Y., Abrol R., Mak J.Y.W., Das Gupta K., Ramnath D., Karunakaran D., Fairlie D.P., Sweet M.J. (2022). Histone deacetylase 7: A signalling hub controlling development, inflammation, metabolism and disease.. FEBS J..

[59] Jiao F., Gong Z. (2020). The beneficial roles of SIRT1 in neuroinflammation-related diseases.. Oxid. Med. Cell. Longev..

[60] Kee H.J., Kim I., Jeong M.H. (2022). Zinc-dependent histone deacetylases: Potential therapeutic targets for arterial hypertension.. Biochem. Pharmacol..

[61] Nayak R., Rosh I., Kustanovich I., Stern S. (2021). Mood stabilizers in psychiatric disorders and mechanisms learnt from in vitro model systems.. Int. J. Mol. Sci..

[62] Tasneem S., Alam M.M., Amir M., Akhter M., Parvez S., Verma G., Nainwal L.M., Equbal A., Anwer T., Shaquiquzzaman M. (2022). Heterocyclic Moieties as HDAC Inhibitors: Role in cancer therapeutics.. Mini Rev. Med. Chem..

[63] Singh A., Bishayee A., Pandey A. (2018). Targeting histone deacetylases with natural and synthetic agents: An emerging anticancer strategy.. Nutrients.

[64] Eckschlager T., Plch J., Stiborova M., Hrabeta J. (2017). Histone deacetylase inhibitors as anticancer drugs.. Int. J. Mol. Sci..

[65] He J., Chu Y., Li J., Meng Q., Liu Y., Jin J., Wang Y., Wang J., Huang B., Shi L., Shi X., Tian J., Zhufeng Y., Feng R., Xiao W., Gan Y., Guo J., Shao C., Su Y., Hu F., Sun X., Yu J., Kang Y., Li Z. (2022). Intestinal butyrate-metabolizing species contribute to autoantibody production and bone erosion in rheumatoid arthritis.. . Sci. Adv.,.

[66] Mazzocchi M., Goulding S.R., Morales-Prieto N., Foley T., Collins L.M., Sullivan A.M., O’Keeffe G.W. (2022). Peripheral administration of the Class-IIa HDAC inhibitor MC1568 partially protects against nigrostriatal neurodegeneration in the striatal 6-OHDA rat model of Parkinson’s disease.. Brain Behav. Immun..

[67] Brookes R.L., Crichton S., Wolfe C.D.A., Yi Q., Li L., Hankey G.J., Rothwell P.M., Markus H.S. (2018). Sodium valproate, a histone deacetylase inhibitor, Is associated with reduced stroke risk after previous ischemic stroke or transient ischemic attack.. Stroke.

[68] Gupta R., Ambasta R.K., Kumar P. (2021). Histone deacetylase in neuropathology.. Adv. Clin. Chem..

[69] Kumar S., Attrish D., Srivastava A., Banerjee J., Tripathi M., Chandra P.S., Dixit A.B. (2021). Non-histone substrates of histone deacetylases as potential therapeutic targets in epilepsy.. Expert Opin. Ther. Targets.

[70] Wang G., Jiang X., Pu H., Zhang W., An C., Hu X., Liou A.K.F., Leak R.K., Gao Y., Chen J. (2013). Scriptaid, a novel histone deacetylase inhibitor, protects against traumatic brain injury via modulation of PTEN and AKT pathway: scriptaid protects against TBI via AKT.. Neurotherapeutics.

[71] Wang G., Shi Y., Jiang X., Leak R.K., Hu X., Wu Y., Pu H., Li W.W., Tang B., Wang Y., Gao Y., Zheng P., Bennett M.V.L., Chen J. (2015). HDAC inhibition prevents white matter injury by modulating microglia/macrophage polarization through the GSK3β/PTEN/Akt axis.. Proc. Natl. Acad. Sci. USA.

[72] Meng Q., Yang G., Yang Y., Ding F., Hu F. (2020). Protective effects of histone deacetylase inhibition by Scriptaid on brain injury in neonatal rat models of cerebral ischemia and hypoxia.. Int. J. Clin. Exp. Pathol..

[73] Chang P., Williams A.M., Bhatti U.F., Biesterveld B.E., Liu B., Nikolian V.C., Dennahy I.S., Lee J., Li Y., Alam H.B. (2019). Valproic acid and neural apoptosis, inflammation, and degeneration 30 days after traumatic brain injury, hemorrhagic shock, and polytrauma in a swine model.. J. Am. Coll. Surg..

[74] Bambakidis T., Dekker S.E., Sillesen M., Liu B., Johnson C.N., Jin G., de Vries H.E., Li Y., Alam H.B. (2016). Resuscitation with valproic acid alters inflammatory genes in a porcine model of combined traumatic brain injury and hemorrhagic shock.. J. Neurotrauma.

[75] Wakam G.K., Biesterveld B.E., Pai M.P., Kemp M.T., O’Connell R.L., Williams A.M., Srinivasan A., Chtraklin K., Siddiqui A.Z., Bhatti U.F., Vercruysse C.A., Alam H.B. (2021). Administration of valproic acid in clinically approved dose improves neurologic recovery and decreases brain lesion size in swine subjected to hemorrhagic shock and traumatic brain injury.. J. Trauma Acute Care Surg..

[76] Dash P.K., Orsi S.A., Zhang M., Grill R.J., Pati S., Zhao J., Moore A.N. (2010). Valproate administered after traumatic brain injury provides neuroprotection and improves cognitive function in rats.. PLoS One.

[77] Bhatti U.F., Karnovsky A., Dennahy I.S., Kachman M., Williams A.M., Nikolian V.C., Biesterveld B.E., Siddiqui A., O’Connell R.L., Liu B., Li Y., Alam H.B. (2021). Pharmacologic modulation of brain metabolism by valproic acid can induce a neuroprotective environment.. J. Trauma Acute Care Surg..

[78] Jepsen C.H., deMoya M.A., Perner A., Sillesen M., Ostrowski S.R., Alam H.B., Johansson P.I. (2014). Effect of valproic acid and injury on lesion size and endothelial glycocalyx shedding in a rodent model of isolated traumatic brain injury.. J. Trauma Acute Care Surg..

[79] Dekker S.E., Bambakidis T., Sillesen M., Liu B., Johnson C.N., Jin G., Li Y., Alam H.B. (2014). Effect of pharmacologic resuscitation on the brain gene expression profiles in a swine model of traumatic brain injury and hemorrhage.. J. Trauma Acute Care Surg..

[80] Dekker S.E., Biesterveld B.E., Bambakidis T., Williams A.M., Tagett R., Johnson C.N., Sillesen M., Liu B., Li Y., Alam H.B. (2021). modulation of brain transcriptome by combined histone deacetylase inhibition and plasma treatment following traumatic brain injury and hemorrhagic shock.. Shock.

[81] Weykamp M., Nikolian V.C., Dennahy I.S., Higgins G.A., Georgoff P.E., Remmer H., Ghandour M.H., Alam H.B. (2018). Rapid valproic acid-induced modulation of the traumatic proteome in a porcine model of traumatic brain injury and hemorrhagic shock.. J. Surg. Res..

[82] Shein N.A., Grigoriadis N., Alexandrovich A.G., Simeonidou C., Lourbopoulos A., Polyzoidou E., Trembovler V., Mascagni P., Dinarello C.A., Shohami E. (2009). Histone deacetylase inhibitor ITF2357 is neuroprotective, improves functional recovery, and induces glial apoptosis following experimental traumatic brain injury.. FASEB J..

[83] Sagarkar S., Balasubramanian N., Mishra S., Choudhary A.G., Kokare D.M., Sakharkar A.J. (2019). Repeated mild traumatic brain injury causes persistent changes in histone deacetylase function in hippocampus: Implications in learning and memory deficits in rats.. Brain Res..

[84] Li T., Zhang Y., Han D., Hua R., Guo B., Hu S., Yan X., Xu T. (2017). Involvement of IL-17 in secondary brain injury after a traumatic brain injury in rats.. Neuromol. Med..

[85] Xu J., Shi J., Zhang J., Zhang Y. (2018). Vorinostat: a histone deacetylases (HDAC) inhibitor ameliorates traumatic brain injury by inducing iNOS/Nrf2/ARE pathway.. Folia Neuropathol..

[86] Balasubramanian N., Sagarkar S., Jadhav M., Shahi N., Sirmaur R., Sakharkar A.J. (2021). Role for histone deacetylation in traumatic brain injury-induced deficits in neuropeptide y in arcuate nucleus: Possible implications in feeding behavior.. Neuroendocrinology.

[87] Dash P.K., Orsi S.A., Moore A.N. (2009). Histone deactylase inhibition combined with behavioral therapy enhances learning and memory following traumatic brain injury.. Neuroscience.

[88] Nikolian V.C., Dennahy I.S., Weykamp M., Williams A.M., Bhatti U.F., Eidy H., Ghandour M.H., Chtraklin K., Li Y., Alam H.B. (2019). Isoform 6–selective histone deacetylase inhibition reduces lesion size and brain swelling following traumatic brain injury and hemorrhagic shock.. J. Trauma Acute Care Surg..

[89] Zhang B., West E.J., Van K.C., Gurkoff G.G., Zhou J., Zhang X.M., Kozikowski A.P., Lyeth B.G. (2008). HDAC inhibitor increases histone H3 acetylation and reduces microglia inflammatory response following traumatic brain injury in rats.. Brain Res..

[90] Dekker S.E., Sillesen M., Bambakidis T., Andjelkovic A.V., Jin G., Liu B., Boer C., Johansson P.I., Linzel D., Halaweish I., Alam H.B. (2014). Treatment with a histone deacetylase inhibitor, valproic acid, is associated with increased platelet activation in a large animal model of traumatic brain injury and hemorrhagic shock.. J. Surg. Res..

[91] Yu F., Wang Z., Tanaka M., Chiu C.T., Leeds P., Zhang Y., Chuang D.M. (2013). Posttrauma cotreatment with lithium and valproate: reduction of lesion volume, attenuation of blood-brain barrier disruption, and improvement in motor coordination in mice with traumatic brain injury.. J. Neurosurg..

[92] Wang W., Tan T., Cao Q., Zhang F., Rein B., Duan W.M., Yan Z. (2022). Histone deacetylase inhibition restores behavioral and synaptic function in a mouse model of 16p11.2 Deletion.. Int. J. Neuropsychopharmacol..

[93] Kusaczuk M., Krętowski R., Stypułkowska A., Cechowska-Pasko M. (2016). Molecular and cellular effects of a novel hydroxamate-based HDAC inhibitor – belinostat – in glioblastoma cell lines: a preliminary report.. Invest. New Drugs.

[94] Rodríguez-Gómez J.A., Kavanagh E., Engskog-Vlachos P., Engskog M.K.R., Herrera A.J., Espinosa-Oliva A.M., Joseph B., Hajji N., Venero J.L., Burguillos M.A. (2020). Microglia: Agents of the CNS Pro-inflammatory response.. Cells.

[95] Yadav A., Huang T.C., Chen S.H., Ramasamy T.S., Hsueh Y.Y., Lin S.P., Lu F.I., Liu Y.H., Wu C.C. (2021). Sodium phenylbutyrate inhibits Schwann cell inflammation via HDAC and NFκB to promote axonal regeneration and remyelination.. J. Neuroinflammation.

[96] Cho W., Hong S.H., Choe J. (2013). IL-4 and HDAC Inhibitors Suppress Cyclooxygenase-2 expression in human follicular dendritic cells.. Immune Netw..

[97] Yang H., Ni W., Wei P., Li S., Gao X., Su J., Jiang H., Lei Y., Zhou L., Gu Y. (2021). HDAC inhibition reduces white matter injury after intracerebral hemorrhage.. J. Cereb. Blood Flow Metab..

[98] Patnala R., Arumugam T.V., Gupta N., Dheen S.T. (2017). HDAC inhibitor sodium butyrate-mediated epigenetic regulation enhances neuroprotective function of microglia during ischemic stroke.. Mol. Neurobiol..

[99] Czapski G.A., Strosznajder J.B. (2021). Glutamate and GABA in microglia-neuron cross-talk in alzheimer’s disease.. Int. J. Mol. Sci..

[100] Nathalie M., Polineni S.P., Chin C.N., Fawcett D., Clervius H., Maria Q.S.L., Legnay F., Rego L., Mahavadi A.K., Jermakowicz W.J., Sw-T L., Yokobori S., Gajavelli S. (2021). Targeting microglial polarization to improve TBI outcomes.. CNS Neurol. Disord. Drug Targets.

[101] Shein N.A., Shohami E. (2011). Histone deacetylase inhibitors as therapeutic agents for acute central nervous system injuries.. Mol. Med..

[102] Glauben R., Siegmund B. (2011). Inhibition of histone deacetylases in inflammatory bowel diseases.. Mol. Med..

[103] Dietz K.C., Casaccia P. (2010). HDAC inhibitors and neurodegeneration: At the edge between protection and damage.. Pharmacol. Res..

[104] Gupta R., Ambasta R.K., Kumar P. (2020). Pharmacological intervention of histone deacetylase enzymes in the neurodegenerative disorders.. Life Sci..

[105] Chen J., Zhang J., Shaik N.F., Yi B., Wei X., Yang X.F., Naik U.P., Summer R., Yan G., Xu X., Sun J. (2019). The histone deacetylase inhibitor tubacin mitigates endothelial dysfunction by up-regulating the expression of endothelial nitric oxide synthase.. J. Biol. Chem..

[106] Shen Y., Yang R., Zhao J., Chen M., Chen S., Ji B., Chen H., Liu D., Li L., Du G. (2022). The histone deacetylase inhibitor belinostat ameliorates experimental autoimmune encephalomyelitis in mice by inhibiting TLR2/MyD88 and HDAC3/NF-κB p65-mediated neuroinflammation.. Pharmacol. Res..

[107] Royce S.G., Dang W., Yuan G., Tran J., El-Osta A., Karagiannis T.C., Tang M.L.K. (2012). Effects of the histone deacetylase inhibitor, trichostatin A, in a chronic allergic airways disease model in mice.. Arch. Immunol. Ther. Exp. (Warsz.).

[108] Dinarello C.A. (2010). Anti-inflammatory agents: Present and future.. Cell.

[109] Khatri N., Thakur M., Pareek V., Kumar S., Sharma S., Datusalia A.K. (2018). Oxidative stress: Major threat in traumatic brain injury.. CNS Neurol. Disord. Drug Targets.

[110] Calabrese V., Cornelius C., Dinkova-Kostova A.T., Calabrese E.J., Mattson M.P. (2010). Cellular stress responses, the hormesis paradigm, and vitagenes: Novel targets for therapeutic intervention in neurodegenerative disorders.. Antioxid. Redox Signal..

[111] Calabrese V., Cornelius C., Dinkova-Kostova A.T., Calabrese E.J. (2009). Vitagenes, cellular stress response, and acetylcarnitine: Relevance to hormesis.. Biofactors.

[112] Calabrese V., Mancuso C., Calvani M., Rizzarelli E., Butterfield D.A., Giuffrida Stella A.M. (2007). Nitric oxide in the central nervous system: Neuroprotection versus neurotoxicity.. Nat. Rev. Neurosci..

[113] Renis M., Calabrese V., Russo A., Calderone A., Barcellona M.L., Rizza V. (1996). Nuclear DNA strand breaks during ethanol-induced oxidative stress in rat brain.. FEBS Lett..

[114] Misztak P., Sowa-Kućma M., Szewczyk B., Nowak G. (2021). Vorinostat (SAHA) may exert its antidepressant-like effects through the modulation of oxidative stress pathways.. Neurotox. Res..

[115] Valvassori S.S., Dal-Pont G.C., Steckert A.V., Varela R.B., Lopes-Borges J., Mariot E., Resende W.R., Arent C.O., Carvalho A.F., Quevedo J. (2016). Sodium butyrate has an antimanic effect and protects the brain against oxidative stress in an animal model of mania induced by ouabain.. Psychiatry Res..

[116] Varoglu A.O., Yildirim A., Aygul R., Gundogdu O.L., Sahin Y.N. (2010). Effects of valproate, carbamazepine, and levetiracetam on the antioxidant and oxidant systems in epileptic patients and their clinical importance.. Clin. Neuropharmacol..

[117] Fu J., Shao C.J., Chen F.R., Ng H.K., Chen Z.P. (2010). Autophagy induced by valproic acid is associated with oxidative stress in glioma cell lines.. Neuro-oncol..

[118] Fourcade S., Ruiz M., Guilera C., Hahnen E., Brichta L., Naudi A., Portero-Otín M., Dacremont G., Cartier N., Wanders R., Kemp S., Mandel J.L., Wirth B., Pamplona R., Aubourg P., Pujol A. (2010). Valproic acid induces antioxidant effects in X-linked adrenoleukodystrophy.. Hum. Mol. Genet..

[119] Iranpak F., Saberzadeh J., Vessal M., Takhshid M.A. (2019). Sodium valproate ameliorates aluminum-induced oxidative stress and apoptosis of PC12 cells.. Iran. J. Basic Med. Sci..

[120] Sun X., Sun Y., Lin S., Xu Y., Zhao D. (2020). Histone deacetylase inhibitor valproic acid attenuates high glucose induced endoplasmic reticulum stress and apoptosis in NRK 52E cells.. Mol. Med. Rep..

[121] Wu M.S., Li X.J., Liu C.Y., Xu Q., Huang J.Q., Gu S., Chen J.X. (2022). Effects of histone modification in major depressive disorder.. Curr. Neuropharmacol..

[122] Faraco G., Pancani T., Formentini L., Mascagni P., Fossati G., Leoni F., Moroni F., Chiarugi A. (2006). Pharmacological inhibition of histone deacetylases by suberoylanilide hydroxamic acid specifically alters gene expression and reduces ischemic injury in the mouse brain.. Mol. Pharmacol..

[123] Lee H.A., Lee E., Do G.Y., Moon E.K., Quan F.S., Kim I. (2019). Histone deacetylase inhibitor MGCD0103 protects the pancreas from streptozotocin-induced oxidative stress and β-cell death.. Biomed. Pharmacother..

[124] Langley B., Gensert J., Beal M., Ratan R. (2005). Remodeling chromatin and stress resistance in the central nervous system: histone deacetylase inhibitors as novel and broadly effective neuroprotective agents.. Curr. Drug Targets CNS Neurol. Disord..

[125] Ferrante R.J., Kubilus J.K., Lee J., Ryu H., Beesen A., Zucker B., Smith K., Kowall N.W., Ratan R.R., Luthi-Carter R., Hersch S.M. (2003). Histone deacetylase inhibition by sodium butyrate chemotherapy ameliorates the neurodegenerative phenotype in Huntington’s disease mice.. J. Neurosci..

[126] Graham N.S.N., Sharp D.J. (2019). Understanding neurodegeneration after traumatic brain injury: From mechanisms to clinical trials in dementia.. J. Neurol. Neurosurg. Psychiatry.

[127] Toshkezi G., Kyle M., Longo S.L., Chin L.S., Zhao L.R. (2018). Brain repair by hematopoietic growth factors in the subacute phase of traumatic brain injury.. J. Neurosurg..

[128] Kitahara M., Inoue T., Mani H., Takamatsu Y., Ikegami R., Tohyama H., Maejima H. (2021). Exercise and pharmacological inhibition of histone deacetylase improves cognitive function accompanied by an increase of gene expressions crucial for neuronal plasticity in the hippocampus.. Neurosci. Lett..

[129] Pawelec P., Sypecka J., Zalewska T., Ziemka-Nalecz M. (2022). Analysis of Givinostat/ITF2357 treatment in a rat model of neonatal hypoxic-ischemic brain damage.. Int. J. Mol. Sci..

[130] Francelle L., Outeiro T.F., Rappold G.A. (2020). Inhibition of HDAC6 activity protects dopaminergic neurons from alpha-synuclein toxicity.. Sci. Rep..

[131] Gao W.M., Chadha M.S., Kline A.E., Clark R.S.B., Kochanek P.M., Dixon C.E., Jenkins L.W. (2006). Immunohistochemical analysis of histone H3 acetylation and methylation—Evidence for altered epigenetic signaling following traumatic brain injury in immature rats.. Brain Res..

[132] Guan J.S., Haggarty S.J., Giacometti E., Dannenberg J.H., Joseph N., Gao J., Nieland T.J.F., Zhou Y., Wang X., Mazitschek R., Bradner J.E., DePinho R.A., Jaenisch R., Tsai L.H. (2009). HDAC2 negatively regulates memory formation and synaptic plasticity.. Nature.

[133] Prior R., Van Helleputte L., Klingl Y.E., Van Den Bosch L. (2018). HDAC6 as a potential therapeutic target for peripheral nerve disorders.. Expert Opin. Ther. Targets.

[134] Calliari A., Bobba N., Escande C., Chini E.N. (2014). Resveratrol delays Wallerian degeneration in a NAD+ and DBC1 dependent manner.. Exp. Neurol..

[135] Zhan X., Cox C., Ander B.P., Liu D., Stamova B., Jin L.W., Jickling G.C., Sharp F.R. (2015). Inflammation combined with ischemia produces myelin injury and plaque-like aggregates of myelin, amyloid-β and AβPP in adult rat brain.. J. Alzheimers Dis..

[136] Xu Z., Lv X.A., Dai Q., Ge Y.Q., Xu J. (2016). Acute upregulation of neuronal mitochondrial type-1 cannabinoid receptor and it’s role in metabolic defects and neuronal apoptosis after TBI.. Mol. Brain.

[137] Buyandelger B., Bar E.E., Hung K.S., Chen R.M., Chiang Y.H., Liou J.P., Huang H.M., Wang J.Y. (2020). Histone deacetylase inhibitor MPT0B291 suppresses glioma growth in vitro and in vivo partially through acetylation of p53.. Int. J. Biol. Sci..

[138] Uo T., Veenstra T.D., Morrison R.S. (2009). Histone deacetylase inhibitors prevent p53-dependent and p53-independent Bax-mediated neuronal apoptosis through two distinct mechanisms.. J. Neurosci..

[139] Cope E.C., Gould E. (2019). Adult neurogenesis, glia, and the extracellular matrix.. Cell Stem Cell.

[140] Nieto-Estevez V., Changarathil G., Adeyeye A.O., Coppin M.O., Kassim R.S., Zhu J., Hsieh J. (2022). HDAC1 regulates neuronal differentiation.. Front. Mol. Neurosci..

[141] Yoo D.Y., Kim D.W., Kim M.J., Choi J.H., Jung H.Y., Nam S.M., Kim J.W., Yoon Y.S., Choi S.Y., Hwang I.K. (2015). Sodium butyrate, a histone deacetylase Inhibitor, ameliorates SIRT2-induced memory impairment, reduction of cell proliferation, and neuroblast differentiation in the dentate gyrus.. Neurol. Res..

[142] Uittenbogaard M., Brantner C.A., Chiaramello A. (2018). Epigenetic modifiers promote mitochondrial biogenesis and oxidative metabolism leading to enhanced differentiation of neuroprogenitor cells.. Cell Death Dis..

[143] Moon B.S., Lu W., Park H.J. (2018). Valproic acid promotes the neuronal differentiation of spiral ganglion neural stem cells with robust axonal growth.. Biochem. Biophys. Res. Commun..

[144] Wu C.H., Tsai Y.C., Tsai T.H., Kuo K.L., Su Y.F., Chang C.H., Lin C.L. (2021). Valproic acid reduces vasospasm through modulation of Akt phosphorylation and attenuates neuronal apoptosis in subarachnoid hemorrhage rats.. Int. J. Mol. Sci..

[145] Yu I.T., Park J.Y., Kim S.H., Lee J., Kim Y.S., Son H. (2009). Valproic acid promotes neuronal differentiation by induction of proneural factors in association with H4 acetylation.. Neuropharmacology.

[146] Rao T., Wu F., Xing D., Peng Z., Ren D., Feng W., Chen Y., Zhao Z., Wang H., Wang J., Kan W., Zhang Q. (2014). Effects of valproic Acid on axonal regeneration and recovery of motor function after peripheral nerve injury in the rat.. Arch. Bone Jt. Surg..

[147] Rozenbaum M., Rajman M., Rishal I., Koppel I., Koley S., Medzihradszky K.F., Oses-Prieto J.A., Kawaguchi R., Amieux P.S., Burlingame A.L., Coppola G., Fainzilber M. (2018). Translatome regulation in neuronal injury and axon regrowth. eNeuro,.

[148] Petrova V., Eva R. (2018). The virtuous cycle of axon growth: Axonal transport of growth-promoting machinery as an intrinsic determinant of axon regeneration.. Dev. Neurobiol..

[149] Mahgoub M., Monteggia L.M. (2014). A role for histone deacetylases in the cellular and behavioral mechanisms underlying learning and memory.. Learn. Mem..

[150] Fischer A., Sananbenesi F., Wang X., Dobbin M., Tsai L.H. (2007). Recovery of learning and memory is associated with chromatin remodelling.. Nature.

[151] Gaub P., Tedeschi A., Puttagunta R., Nguyen T., Schmandke A., Di Giovanni S. (2010). HDAC inhibition promotes neuronal outgrowth and counteracts growth cone collapse through CBP/p300 and P/CAF-dependent p53 acetylation.. Cell Death Differ..

[152] Johnson E.C.B., Dammer E.B., Duong D.M., Ping L., Zhou M., Yin L., Higginbotham L.A., Guajardo A., White B., Troncoso J.C., Thambisetty M., Montine T.J., Lee E.B., Trojanowski J.Q., Beach T.G., Reiman E.M., Haroutunian V., Wang M., Schadt E., Zhang B., Dickson D.W., Ertekin-Taner N., Golde T.E., Petyuk V.A., De Jager P.L., Bennett D.A., Wingo T.S., Rangaraju S., Hajjar I., Shulman J.M., Lah J.J., Levey A.I., Seyfried N.T. (2020). Large-scale proteomic analysis of Alzheimer’s disease brain and cerebrospinal fluid reveals early changes in energy metabolism associated with microglia and astrocyte activation.. Nat. Med..

[153] Shanaki-Bavarsad M., Almolda B., González B., Castellano B. (2022). Astrocyte-targeted overproduction of IL-10 reduces neurodegeneration after TBI.. Exp. Neurobiol..

[154] Liu Z., Chopp M. (2016). Astrocytes, therapeutic targets for neuroprotection and neurorestoration in ischemic stroke.. Prog. Neurobiol..

[155] Wang J., Hou Y., Zhang L., Liu M., Zhao J., Zhang Z., Ma Y., Hou W. (2021). Estrogen attenuates traumatic brain injury by inhibiting the activation of microglia and astrocyte-mediated neuroinflammatory responses.. Mol. Neurobiol..

[156] Borgonetti V., Meacci E., Pierucci F., Romanelli M.N., Galeotti N. (2022). Dual HDAC/BRD4 inhibitors relieves neuropathic pain by attenuating inflammatory response in microglia after spared nerve injury.. Neurotherapeutics.

[157] Prozorovski T., Schulze-Topphoff U., Glumm R., Baumgart J., Schröter F., Ninnemann O., Siegert E., Bendix I., Brüstle O., Nitsch R., Zipp F., Aktas O. (2008). Sirt1 contributes critically to the redox-dependent fate of neural progenitors.. Nat. Cell Biol..

[158] Zhang Y., Du Z., Zhuang Z., Wang Y., Wang F., Liu S., Wang H., Feng H., Li H., Wang L., Zhang X., Hao A. (2015). E804 induces growth arrest, differentiation and apoptosis of glioblastoma cells by blocking Stat3 signaling.. J. Neurooncol..

[159] Michinaga S., Koyama Y. (2021). Pathophysiological responses and roles of astrocytes in traumatic brain injury.. Int. J. Mol. Sci..

[160] Li X., Su X., Liu R., Pan Y., Fang J., Cao L., Feng C., Shang Q., Chen Y., Shao C., Shi Y. (2021). HDAC inhibition potentiates anti-tumor activity of macrophages and enhances anti-PD-L1-mediated tumor suppression.. Oncogene.

[161] Dong Z., Yang Y., Liu S., Lu J., Huang B., Zhang Y. (2018). HDAC inhibitor PAC-320 induces G2/M cell cycle arrest and apoptosis in human prostate cancer.. Oncotarget.

[162] Dashwood R., Ho E. (2007). Dietary histone deacetylase inhibitors: From cells to mice to man.. Semin. Cancer Biol..

[163] Jaworska J., Zalewska T., Sypecka J., Ziemka-Nalecz M. (2019). Effect of the HDAC inhibitor, sodium butyrate, on neurogenesis in a rat model of neonatal hypoxia–ischemia: Potential mechanism of action.. Mol. Neurobiol..

[164] Tung B., Ma D., Wang S., Oyinlade O., Laterra J., Ying M., Lv S.Q., Wei S., Xia S. (2018). Krüppel-like factor 9 and histone deacetylase inhibitors synergistically induce cell death in glioblastoma stem-like cells.. BMC Cancer.

